# Integration of continuous lumbar drainage and third-generation EGFR-TKI in managing leptomeningeal metastasis-induced life-threatening intracranial hypertension: a case report

**DOI:** 10.3389/fonc.2025.1581754

**Published:** 2025-05-30

**Authors:** Lei Wang, Nianjun Ren, Zhi Tang, Rui Wang, Zhengwen He

**Affiliations:** ^1^ Department of Neurosurgery, Hunan Cancer Hospital and the Affiliated Cancer Hospital of Xiangya School of Medicine, Central South University. Changsha, Hunan, China; ^2^ Hunan Provincial University Key Laboratory of the Fundamental and Clinical Research on Neurodegenerative Diseases, Changsha Medical University, Changsha, Hunan, China

**Keywords:** leptomeningeal metastasis, lumbar drainage, third-generation EGFR-TKI, NSCLC, intracranial hypertension

## Abstract

Leptomeningeal metastasis (LM) from lung cancer carries an extremely poor prognosis, with patients often presenting severe intracranial hypertension symptoms such as intractable headache and recurrent vomiting. Ventriculoperitoneal (VP) shunt placement is commonly used to manage intracranial hypertension in leptomeningeal metastasis but carries risks such as infection, shunt malfunction, tumor seeding, abdominal adhesions, or overdrainage. For patients with suspected EGFR L858R/T790M mutations, lumbar cistern drainage offers a safer alternative by draining cerebrospinal fluid (CSF), reducing intracranial pressure (ICP), and preventing sudden death from critical ICP elevation. This approach also provides a critical therapeutic window for EGFR-TKI therapy. Compared to VP shunts, lumbar cistern drainage is preferred due to its minimally invasive nature, fewer procedural complications, and avoidance of general anesthesia. This study reports a case of EGFR L858R/T790M mutation-positive lung adenocarcinoma with LM and life-threatening intracranial hypertension that achieved marked clinical improvement through combined lumbar drainage and furmonertinib therapy. The approach not only facilitated rapid symptom relief and molecular confirmation of EGFR mutation but also enabled sustained disease control.

## Introduction

Lung cancer remains one of the most prevalent malignancies worldwide, with leptomeningeal metastasis(LM) representing a devastating complication that significantly worsens prognosis (1, 2). LM occurs in approximately 3-5% of non-small-cell lung cancer (NSCLC) patients (3), particularly in those with adenocarcinoma and specific driver mutations such as EGFR and ALK rearrangements (4, 5). LM has a poor prognosis as the life expectancy after diagnosis is 4–6 weeks without treatment (6, 7). Despite advancements in systemic therapies, LM is associated with poor survival, with a median overall survival (OS) ranging from 3 to 11 months, depending on the molecular profile and treatment modalities employed (1–3). Current therapeutic strategies for LM include radiotherapy, systemic and intrathecal chemotherapy, targeted therapies, and immunotherapy, yet the management remains challenging due to the blood-brain barrier and the heterogeneity of the disease (8).

A critical consequence of LM is malignant intracranial hypertension, which is a leading cause of mortality in these patients. intracranial hypertension manifests with severe symptoms such as headaches, nausea, vomiting, and neurological deficits, often requiring urgent intervention (9). The treatment of LM-associated intracranial hypertension is further complicated by the need to balance symptomatic relief with disease-modifying therapies. Effective management requires a multidisciplinary approach, combining immediate measures to reduce intracranial pressure (ICP) with strategies to target the metastatic disease.

Recent clinical studies have demonstrated that in patients with LM, ventriculoperitoneal shunt(VPS) placement not only alleviates intracranial hypertension-related symptoms but also prevents sudden death in those with severe intracranial hypertension. However, while VPS alone does not improve overall survival (OS), it reduces mortality risk attributable to refractory intracranial hypertension. When combined with systemic therapy (e.g., targeted/immunotherapy), LM lesions can be effectively controlled, leading to significant survival prolongation and improved quality of life (10). A recent clinical study highlights that the use of VPS and Ommaya reservoir not only alleviates leptomeningeal metastasis (LM)-induced hydrocephalus but also enables intraventricular chemotherapy delivery via the Ommaya system, significantly improving patients’ quality of life and survival outcomes (11). However, VPS placement carries significant risks in patients with LM, including hemorrhage, infection, device malfunction, and the potential for peritoneal carcinomatosis due to CSF diversion (12, 13).

EGFR-TKIs and ALK-TKIs have shown promising CNS penetration and efficacy in LM patients with corresponding driver mutations, offering a potential avenue to improve outcomes (14–18). Due to their superior CNS penetration and intracranial tumor control efficacy, third-generation EGFR-TKIs are preferred for patients with LM and intracranial hypertension who harbor suspected EGFR mutations. In such cases, clinicians may opt for a less invasive lumbar puncture drainage to stabilize patients during the acute phase of malignant intracranial hypertension, avoiding the need for more aggressive interventions. Compared to VPS placement, lumbar puncture drainage is a minimally invasive procedure that is cost-effective, avoids permanent catheter placement, and eliminates risks associated with VPS such as hemorrhage, overdrainage, and catheter occlusion. Lumbar puncture plays a dual role in this context: it not only provides symptomatic relief by reducing ICP but also enables the collection of cerebrospinal fluid (CSF) for molecular profiling, which is crucial for guiding targeted therapies (9).

## Case report

A 59-year-old male patient was admitted to the hospital with a one-month history of headache, vomiting, and weakness in the right limb. The patient had undergone a left lower lobectomy at Peking University First Hospital in April 2018, with postoperative pathology confirming adenocarcinoma of the lung. Subsequent next-generation sequencing (NGS) of the biopsy tissue revealed a positive result for Exon21 (L858R) mutation, while Exon19 (E746-A750) and ALK protein were negative. Following surgery, the patient received targeted therapy with Tarceva and chemotherapy.

**Figure 1:**
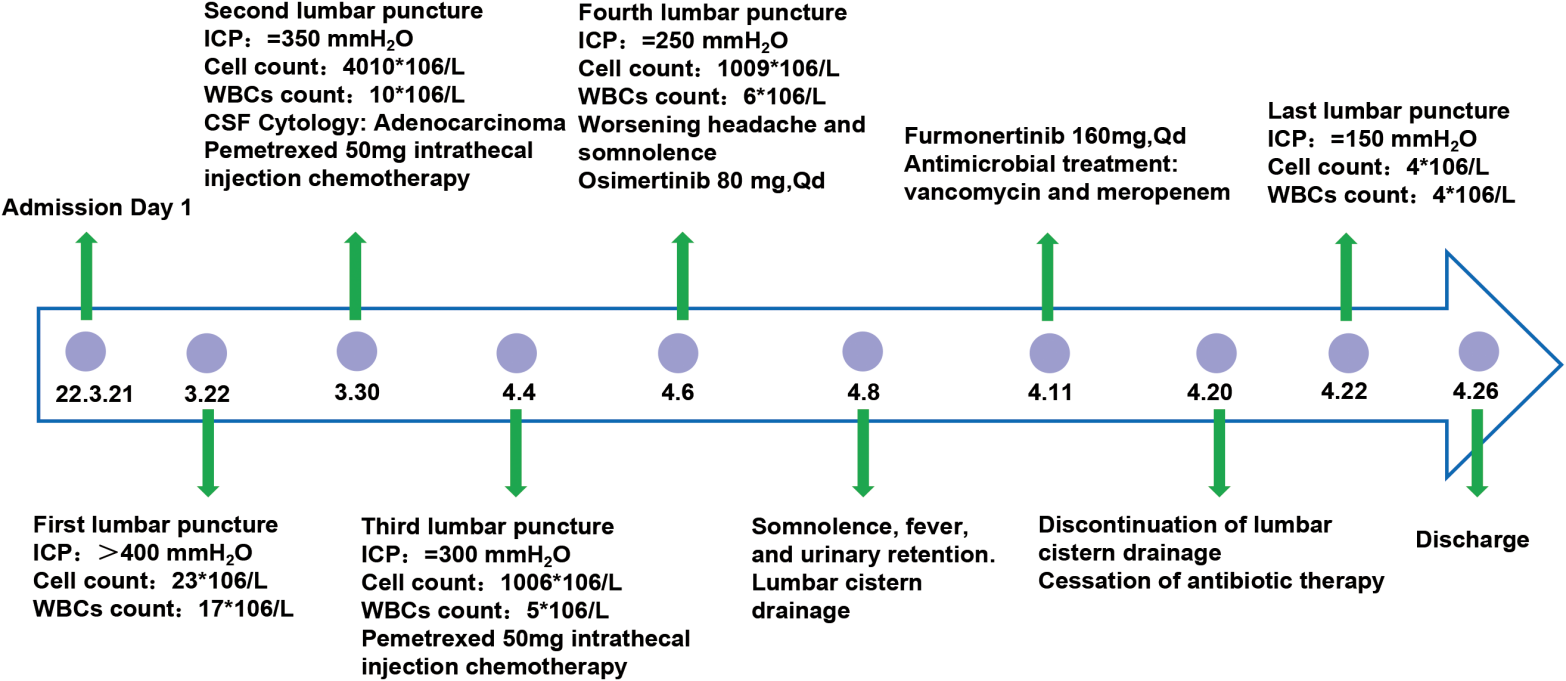
Patients’ admission treatment flowchart.

Upon admission, the patient experienced severe headache accompanied by frequent vomiting. These symptoms were initially misattributed to chemotherapy-related complications and managed symptomatically for two weeks prior to accurate diagnosis. Ultimately, the patient was transferred to the neurosurgery department where osmotic therapy combined with glucocorticoids was administered for intracranial pressure reduction. However, this regimen yielded limited therapeutic efficacy, failing to achieve significant alleviation of the patient’s persistent headache and intractable vomiting. Further PET-MRI imaging revealed bilateral multiple ischemic lesions in the frontal, parietal, and occipital lobes, with no evidence of brain or meningeal metastases. The patient exhibits positive meningeal irritation signs. First lumbar puncture findings: Despite mannitol infusion, ICP remained elevated at >400 mmH_2_O. CSF analysis revealed a total cell count of 23×10^6^/L (23,000/μL), with 17×10^6^/L (17,000/μL) white blood cells (WBCs). Biochemical testing demonstrated elevated glucose levels and decreased protein concentrations (**Figure 1**). Cancer cells were detected in the cerebrospinal fluid (CSF) cytology. Given the potential for genetic differences between the CSF and primary cancer, NGS was conducted on the CSF. The CSF molecular profiling revealed positivity for the EGFR Exon21 (L858R) mutation, concordant with the molecular profile of the primary pulmonary lesion.

Upon diagnosis of leptomeningeal metastases, the patient was treated with intrathecal pemetrexed disodium(50 mg, twice weekly) in combination with temozolomide (150 mg/m²). Despite these interventions, the patient’s symptoms of headache and vomiting persisted, eventually progressing to somnolence. To alleviate intracranial hypertension, a lumbar cisternal shunt was performed. Postoperatively, the patient’s headache and vomiting resolved, and his consciousness improved.

Continuous lumbar puncture for CSF drainage and subsequent lumbar cistern drainage significantly alleviated headache and vomiting symptoms induced by intracranial hypertension, while also providing a therapeutic window for targeted therapy. Surprisingly, NGS of the CSF revealed an EGFR L858R/T790M mutation. Based on these findings, the treatment was switched to osimertinib (80 mg daily). However, the patient developed significant reductions in red blood cells, white blood cells, and platelets following osimertinib administration. Additionally, prolonged lumbar cisternal drainage led to symptoms of intracranial infection. After consultation with the Neuro-Oncology MDT team and experts from Beijing Tiantan Hospital, the treatment was adjusted to furmonertinib(160 mg daily), while continuing lumbar cisternal drainage and administering meropenem plus vancomycin for the intracranial infection. Within three days, the patient’s CSF drainage decreased, fever was controlled, and blood counts returned to near-normal levels. The lumbar cisternal drain was removed after 12 days of treatment. Final lumbar puncture findings: ICP normalized to 150 mmH_2_O. CSF analysis revealed a total cell count of 9×10^6^/L (9,000/μL), with 8×10^6^/L (8,000/μL) WBCs. Biochemical parameters, including glucose and protein levels, returned to normal ranges. After two weeks of furmonertinib therapy, all symptoms resolved.

**Figure 2:**
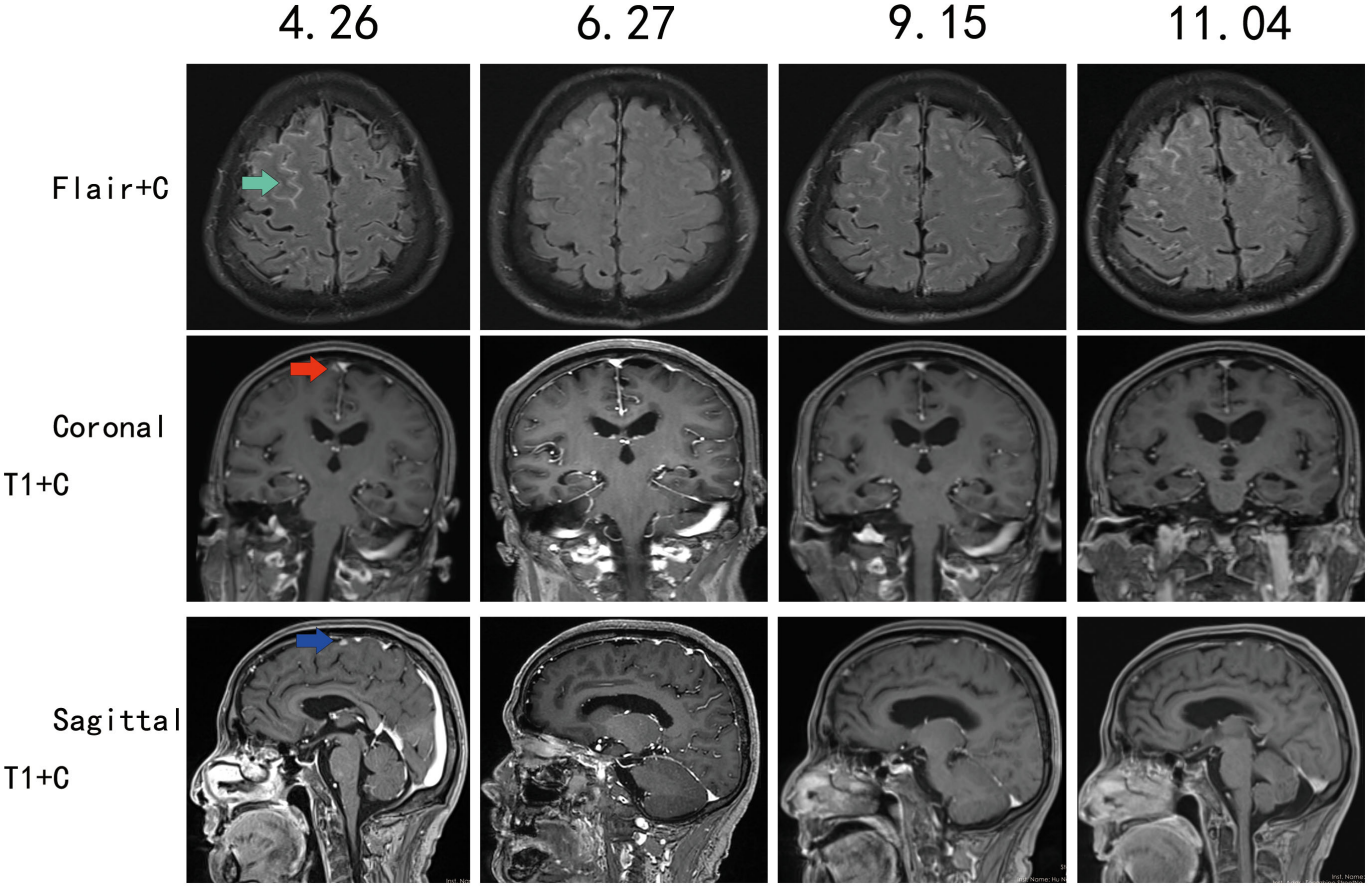
Contrast-enhanced MRI follow-up of the leptomeninges, with follow-up dates on (April 26, June 27, September 15, November 4).

Pre-discharge leptomeningeal MRI (2022 April 26): FLAIR enhancement showed linear leptomeningeal enhancement in the right frontal lobe. Following two months of furmonertinib treatment, the enhancing lesions demonstrated marked diminishment. Follow-up leptomeningeal MRI on September 15 revealed progression of the right frontal lobe meningeal enhancements. Repeat imaging on November 4​showed further significant enlargement of the enhancing lesions compared to the prior study (**Figure 2**). T1-weighted post-contrast imaging on April 26 demonstrated nodular enhancement in the right frontal lobe leptomeninges. This nodular enhancement decreased substantially after treatment and persisted until the final follow-up on November 4 (**Figure 2**).

The patient continued furmonertinib treatment post-discharge, with no recurrence of headache or vomiting, and improved consciousness. The patient reported decreased visual acuity, attributed to optic nerve compression from persistent intracranial hypertension. Six months after discharge, the patient developed progressive cognitive impairment, which gradually worsened over time. Subsequently, recurrent headaches and vomiting re-emerged. The patient ultimately succumbed to the disease in January 2023.

## Discussion

LM remains a devastating complication with dismal prognosis, characterized by rapid neurological deterioration and limited survival, often measured in weeks to months. Currently most of the therapeutic strategies including intrathecal chemotherapy (e.g., pemetrexed), WBRT, and immunotherapy, offer limited efficacy and are often palliative (19, 20). Recent advances emphasize multimodal approaches, combining systemic therapies with localized interventions, yet standardized protocols remain elusive.

LM frequently induces refractory intracranial hypertension due to impaired CSF dynamics and tumor infiltration (21–23). The mechanisms remain incompletely elucidated, with potential pathways including: 1) Tumor cell infiltration along the subarachnoid space, forming nodular or linear lesions that obstruct CSF flow. Imaging may reveal linear meningeal enhancement or nodular lesions, accompanied by ventricular dilation; 2) Activation of meningeal immune cells by tumor cells, triggering pro-inflammatory cytokine secretion, which impairs arachnoid granulation absorption function (24). 3) Chronic inflammation-induced fibrosis, where persistent inflammatory signaling promotes meningeal fibrotic scar formation, further obstructing CSF dynamics and exacerbating absorption deficits; 4)Dysregulation of CSF homeostasis due to tumor-induced overproduction by the choroid plexus, coupled with impaired absorption mechanisms, disrupting the balance between CSF secretion and resorption.

Standard interventions include osmotic agents and CSF diversion to alleviate pressure. In clinical practice, shunt procedures such as ventriculoperitoneal (VP) or lumboperitoneal (LP) shunts are utilized to mitigate ICP-related symptoms and improve patient prognosis (25, 26). Ventriculoperitoneal (VP) shunt placement is currently widely used to treat intracranial hypertension caused by leptomeningeal metastasis and has achieved favorable outcomes. The OS of NSCLC patients with the shunt (7.6 months; 95% CI, 5.8–9.4) was significantly prolonged compared to that of NSCLC patients without the shunt (2.3 months; 95% CI, 1.6–3.0) (22). VP shunt combined with intrathecal chemotherapy has been demonstrated to significantly improve OS in LM patients. VS not only significantly alleviates symptoms caused by intracranial hypertension but also avoids sudden death for those with severe intracranial hypertension (10, 11). However, major complications include infection, shunt malfunction, tumor seeding, abdominal adhesions, or overdrainage (22, 27, 28).

For patients with advanced EGFR-mutant NSCLC, third-generation EGFR-TKIs demonstrate superior BBB penetration, achieving therapeutic CSF concentrations rapidly. Therefore, invasive procedures like intrathecal chemotherapy are unnecessary.

The primary therapeutic objective of lumbar cistern drainage is to drain CSF, reduce ICP, and prevent fatal cerebral crises caused by critically elevated ICP. Additionally, it provides a critical therapeutic time window for genetic testing and ensures sufficient cerebrospinal fluid concentrations of targeted therapies. Compared to VP shunt placement, lumbar cistern drainage is preferred in patients with advanced EGFR-mutant (NSCLC due to its minimally invasive nature, fewer procedural complications, and avoidance of general anesthesia.

In our case, the patient presented with severe malignant intracranial hypertension characterized by intense headaches, vomiting, and even somnolence. We applied continuous lumbar cisternal drainage to release CSF, effectively reducing ICP and saving the patient’s life. This intervention also provided a critical time window for the administration of furmonertinib. Although prolonged lumbar cisternal drainage led to a CNS infection, the infection was successfully controlled with the use of vancomycin and meropenem, and the overall benefits outweighed the risks. This case highlights the significant role of continuous lumbar cisternal drainage in managing malignant intracranial hypertension caused by leptomeningeal metastasis from NSCLC. It not only effectively and significantly reduces ICP but also enables the acquisition of pathological and molecular diagnostic information from CSF, thereby creating a crucial time window for further treatment.More importantly, compared to VP and lumboperitoneal LP shunts, lumbar cisternal drainage externally diverts CSF, thereby preventing the risk of tumor cell dissemination. This procedure is simple, effective, and cost-efficient. Once the drainage tube is removed after the resolution of intracranial hypertension symptoms, no permanent implants are left in the patient’s body.

Molecular characterization of LM is paramount, as genetic discordance between primary tumors and CSF/leptomeningeal lesions is common. For instance, EGFR mutations detected in CSF may differ from primary tissue, necessitating CSF-based NGS to identify actionable targets. In our patient, CSF NGS revealed an EGFR L858R/T790M mutation, enabling a switch to furmonertinib, underscoring the necessity of repeated molecular profiling to adapt therapeutic strategies. Furmonertinib demonstrated robust penetration of the blood-brain barrier (BBB), achieving therapeutic drug concentrations in the CSF (29). Clinical benefits, including tumor cell suppression and restoration of CSF circulation, were observed within 3–4 days, leading to significant alleviation of ICH symptoms. Notably, the drug exhibited a favorable safety profile at a dosage of 160 mg/day, with no significant adverse events reported. For patients presenting with malignant ICH as the initial symptom, particularly those in poor general condition or experiencing cerebral crises, prompt clinical intervention is critical.

## Conclusion

Lumbar cisternal drainage is a simple and effective method for obtaining pathological and molecular diagnostic samples in patients with LM, while also providing CSF drainage to alleviate ICH caused by LM. For patients with EGFR L858R/T790M mutations who also present with ICH, the combination of continuous lumbar cisternal drainage and furmonertinib therapy can effectively mitigate life-threatening complications associated with ICH. This integrated approach not only addresses the immediate risks of elevated intracranial pressure but also exerts a positive therapeutic effect on LM, ultimately improving patient prognosis.

## Data Availability

The datasets presented in this study can be found in online repositories. The names of the repository/repositories and accession number(s) can be found in the article.

## References

[1] Le RE Galanis E . Leptomeningeal metastases of solid cancer. Curr Opin Neurol. (2016) 29:797–805. doi: 10.1097/WCO.0000000000000393 27661208

[2] Li YS Jiang BY Yang JJ . Leptomeningeal metastases in patients with NSCLC with EGFR mutations. J Thorac Oncol. (2016) 11:1962–9. doi: 10.1016/j.jtho.2016.06.029 27539328

[3] Remon J Le RE Besse B . Leptomeningeal carcinomatosis in non-small cell lung cancer patients: A continuing challenge in the personalized treatment era. Cancer Treat Rev. (2017) 53:128–37. doi: 10.1016/j.ctrv.2016.12.006 28110254

[4] Mitra D Chen YH Li R . EGFR mutant locally advanced non-small cell lung cancer is at increased risk of brain metastasis. Clin Transl Radiat Oncol. (2019) 18:32–8. doi: 10.1016/j.ctro.2019.06.008 PMC6612652 31341973

[5] Kim DW Mehra R DSWT . Activity and safety of ceritinib in patients with ALK-rearranged non-small-cell lung cancer (ASCEND-1): updated results from the multicentre, open-label, phase 1 trial. Lancet Oncol. (2016) 17:452–63. doi: 10.1016/S1470-2045(15)00614-2 PMC5063047 26973324

[6] Chamberlain MC . Neoplastic meningitis. Oncologist. (2008) 13:967–77. doi: 10.1634/theoncologist.2008-0138 18776058

[7] Gwak HS Lee SH Park WS Shin SH Yoo H Lee SH . Recent advancements of treatment for leptomeningeal carcinomatosis. J Korean Neurosurg Soc. (2015) 58:1–8. doi: 10.3340/jkns.2015.58.1.1 26279806 PMC4534733

[8] Miller DS . Regulation of P-glycoprotein and other ABC drug transporters at the blood-brain barrier. Trends Pharmacol Sci. (2010) 31:246–54. doi: 10.1016/j.tips.2010.03.003 PMC2882496 20417575

[9] Bander ED Yuan M Reiner AS . Cerebrospinal fluid diversion for leptomeningeal metastasis: palliative, procedural and oncologic outcomes. J Neurooncol. (2021) 154:301–13. doi: 10.1007/s11060-021-03827-2 PMC8504535 34406564

[10] Zhao S Sun P Zhang X . Intraventricular medication with or without ventricular shunt for leptomeningeal metastases with different intracranial pressure: A single-center, large-scale retrospective study. World Neurosurg. (2025) 195:123544. doi: 10.1016/j.wneu.2024.12.003 39662621

[11] Huntoon KM Gasco J Glitza OIC Ferguson SD Majd NK McCutcheon IE . Ventriculoperitoneal shunting with an on-off valve for patients with leptomeningeal metastases and intracranial hypertension. Neurooncol Pract. (2024) 11:56–63. doi: 10.1093/nop/npad056 38222058 PMC10785578

[12] Hoffman HJ Hendrick EB Humphreys RP . Metastasis via ventriculoperitoneal shunt in patients with medulloblastoma. J Neurosurg. (1976) 44:562–6. doi: 10.3171/jns.1976.44.5.0562 1262915

[13] Menger RP Connor DE Thakur JD . A comparison of lumboperitoneal and ventriculoperitoneal shunting for idiopathic intracranial hypertension: an analysis of economic impact and complications using the Nationwide Inpatient Sample. Neurosurg Focus. (2014) 37:E4. doi: 10.3171/2014.8.FOCUS14436 25363432

[14] Chen H Yang S Wang L . High-dose furmonertinib in patients with EGFR-mutated NSCLC and leptomeningeal metastases: A prospective real-world study. J Thorac Oncol. (2025) 20:65–75. doi: 10.1016/j.jtho.2024.09.1385 39260521

[15] Wu H Zhang Q Zhai W . Effectiveness of high-dose third-generation EGFR-tyrosine kinase inhibitors in treating EGFR-mutated non-small cell lung cancer patients with leptomeningeal metastasis. Lung Cancer. (2024) 188:107475. doi: 10.1016/j.lungcan.2024.107475 38266613

[16] Jia C Xu Q Zhao L Kong F Jia Y . Therapeutic role of EGFR - Tyrosine kinase inhibitors in non-small cell lung cancer with leptomeningeal metastasis. Transl Oncol. (2024) 39:101832. doi: 10.1016/j.tranon.2023.101832 38006761 PMC10728707

[17] Lavaud P Bortolot M Zullo L . Early-stage non-small cell lung cancer: new challenges with immune checkpoint blockers and targeted therapies. Cancers (Basel). (2024) 16. doi: 10.3390/cancers16162779 PMC11353229 39199552

[18] Zou Z Xing P Hao X . Intracranial efficacy of alectinib in ALK-positive NSCLC patients with CNS metastases-a multicenter retrospective study. BMC Med. (2022) 20:12. doi: 10.1186/s12916-021-02207-x 35039026 PMC8764827

[19] Ozcan G Singh M Vredenburgh JJ . Leptomeningeal metastasis from non-small cell lung cancer and current landscape of treatments. Clin Cancer Res. (2023) 29:11–29. doi: 10.1158/1078-0432.CCR-22-1585 35972437

[20] Wang Y Yang X Li NJ Xue JX . Leptomeningeal metastases in non-small cell lung cancer: Diagnosis and treatment. Lung Cancer. (2022) 174:1–13. doi: 10.1016/j.lungcan.2022.09.013 36206679

[21] Omuro AM Lallana EC Bilsky MH DeAngelis LM . Ventriculoperitoneal shunt in patients with leptomeningeal metastasis. Neurology. (2005) 64:1625–7. doi: 10.1212/01.WNL.0000160396.69050.DC 15883329

[22] Kim HS Park JB Gwak HS Kwon JW Shin SH Yoo H . Clinical outcome of cerebrospinal fluid shunts in patients with leptomeningeal carcinomatosis. World J Surg Oncol. (2019) 17:59. doi: 10.1186/s12957-019-1595-7 30917830 PMC6438037

[23] Nigim F Critchlow JF Kasper EM . Role of ventriculoperitoneal shunting in patients with neoplasms of the central nervous system: An analysis of 59 cases. Mol Clin Oncol. (2015) 3:1381–6. doi: 10.3892/mco.2015.627 PMC4665378 26807251

[24] Zhao J Zeng R Li X . Dura immunity configures leptomeningeal metastasis immunosuppression for cerebrospinal fluid barrier invasion. Nat Cancer. (2024) 5:1940–61. doi: 10.1038/s43018-024-00858-2 39710801

[25] Mitsuya K Nakasu Y Hayashi N . Palliative cerebrospinal fluid shunting for leptomeningeal metastasis-related hydrocephalus in patients with lung adenocarcinoma: A single-center retrospective study. PloS One. (2019) 14:e0210074. doi: 10.1371/journal.pone.0210074 30629621 PMC6328154

[26] Murakami Y Ichikawa M Bakhit M . Palliative shunt surgery for patients with leptomeningeal metastasis. Clin Neurol Neurosurg. (2018) 168:175–8. doi: 10.1016/j.clineuro.2018.03.008 29567579

[27] Berger MS Baumeister B Geyer JR Milstein J Kanev PM LeRoux PD . The risks of metastases from shunting in children with primary central nervous system tumors. J Neurosurg. (1991) 74:872–7. doi: 10.3171/jns.1991.74.6.0872 2033446

[28] Melisko ME Glantz M Rugo HS . New challenges and opportunities in the management of brain metastases in patients with ErbB2-positive metastatic breast cancer. Nat Clin Pract Oncol. (2009) 6:25–33. doi: 10.1038/ncponc1243 18936791

[29] Chen T Chen J Liu DS . Successful therapy using high-dose furmonertinib for non-small cell lung cancer with leptomeningeal metastasis: a case report and literature review. Front Oncol. (2023) 13:1233198. doi: 10.3389/fonc.2023.1233198 37920163 PMC10619657

